# Left atrial myxoma complicated by abdominal aortic embolization: A case report

**DOI:** 10.1016/j.ijscr.2025.112038

**Published:** 2025-10-06

**Authors:** The-May Nguyen, Minh-Son Le, Doan Quoc Hung, Cong-Huy Nguyen, Minh-Tung Do

**Affiliations:** a Department of Surgery, Viet Tiep Friendship Hospital, Hai Phong City, Viet Nam; b Department of Cardiothoracic Surgery, Viet Tiep Friendship Hospital, Hai Phong City, Viet Nam; c Vinmec International Hospital Times City, Ha Noi, Viet Nam; d Vin University, Ha Noi, Viet Nam; e Department of Surgery, Hai Phong University of Medicine and Pharmacy, Hai Phong City, Viet Nam

**Keywords:** Abdominal aortic embolization, Arterial embolism, Atrial myxoma, Cardiac tumor, Left atrium

## Abstract

**Background:**

Abdominal aortic embolization is an exceptionally rare event. While some case reports have noted abdominal aortic aneurysms and occlusions caused by malignancy, abdominal aortic embolism due to a cardiac tumor is unusual. We present a case of infrarenal abdominal aortic embolization resulting from a left atrial myxoma.

**Case presentation:**

A 37-year-old man presented with acute bilateral lower limb symptoms. Imaging revealed occlusion of the infrarenal abdominal aorta and both common iliac arteries. Surgical exploration identified and removed a gelatinous mass consistent with a myxoma. Histopathology confirmed the diagnosis. Postoperative echocardiography initially revealed a small residual stalk in the left atrium, which did not demonstrate further growth on follow-up imaging for up to four years. The patient demonstrated a successful recovery and exhibited no signs of recurrence during subsequent follow-up evaluations.

**Clinical discussion:**

This case underscores the diagnostic challenges in atypical aortic occlusion without ischemic signs and highlights the importance of early cardiac imaging in suspected embolic events. The presence of collateral circulation may obscure classical symptoms. n instances where a residual stalk is observed in the left atrium, close monitoring with serial echocardiography may represent a feasible and appropriate strategy.

**Conclusions:**

Abdominal aortic embolization secondary to atrial myxoma is rare but life-threatening. Prompt diagnosis using CT angiography and echocardiography, followed by timely surgical management, is critical for survival. Long-term follow-up is essential due to the risk of recurrence.

## Highlights

• Abdominal aortic embolization due to atrial myxoma is extremely rare and may present without classical ischemic symptoms.
• Postoperative echocardiography showed a small stalk-like remnant that resolved spontaneously; no surgery was needed.
• This case underscores the role of preoperative echocardiography in patients with unexplained large-vessel embolism.

## 1 Introduction

Acute abdominal aortic embolization is a rare but life-threatening condition. More common causes include abdominal aortic aneurysm [1] or tumor-related embolism following thoracic surgery, particularly lung cancer [2]. Embolization due to cardiac tumors, especially atrial myxoma, is exceptionally rare. Atrial myxoma accounts for approximately 13 % of systemic embolic events, most frequently affecting the brain and peripheral arteries [3]. Embolization to the abdominal aorta is exceedingly uncommon and may present atypically depending on the degree of collateral circulation. We present a rare case of infrarenal abdominal aortic embolization from a left atrial myxoma, emphasizing diagnostic challenges and the potential implications for surgical decision-making. The present case report has been prepared and reported in accordance with the Surgical CAse REport (SCARE) criteria [4].

## 2 Case presentation

A 37-year-old male with no prior cardiovascular disease and a history of chronic hepatitis B infection presented to the emergency department with complaints of bilateral leg pain and numbness for approximately 8 h. Upon examination, femoral pulses were absent bilaterally; however, the legs were warm, non-cyanotic, and well-perfused (SpO2 95 % bilaterally). The electrocardiogram (ECG) demonstrated normal sinus rhythm. Laboratory investigations were largely unremarkable, with the exception of elevated creatine kinase levels (1405.3 U/L). Given the atypical presentation of acute embolization, computed tomography (CT) angiography of the aorta, iliac arteries and lower extremity vascular system was conducted.

**Fig. 1:**
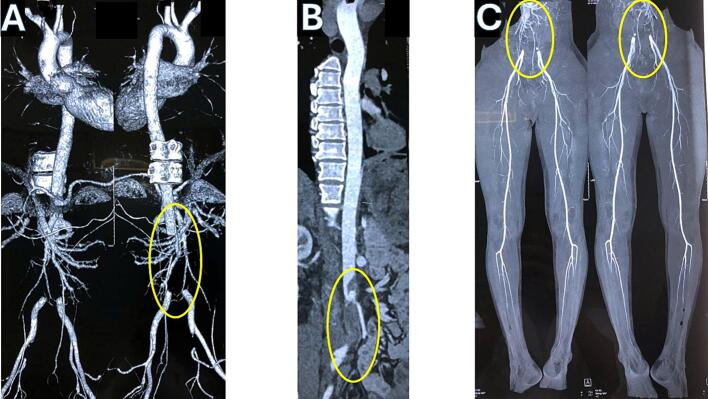
Computed tomography (CT) angiography of the aorta–iliac and lower limb arterial systems. (A-B) Contrast-enhanced CT with 3D reconstruction demonstrates complete occlusion of the infrarenal abdominal aorta and bilateral common iliac arteries (yellow ellipses). (C) In contrast, the lower limb arterial system shows no evidence of obstruction, indicating preserved distal perfusion via collateral circulation. (For interpretation of the references to colour in this figure legend, the reader is referred to the web version of this article.)

Imaging revealed complete occlusion of the abdominal aorta below the inferior mesenteric artery and bilateral common iliac arteries. The vessel walls were smooth without calcification, consistent with an acute embolism (Fig. 1A and B). Interestingly, there was collateral circulation suggestive of some chronicity. The vascular supply to the lower extremities was unobstructed (Fig. 1C).

**Fig. 2:**
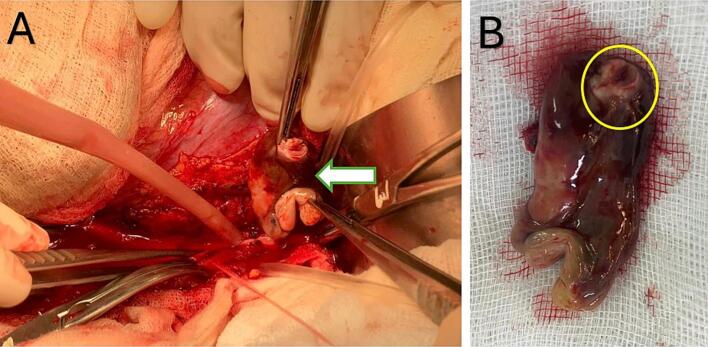
Surgical removal of embolic gelatinous myxoma from the abdominal aorta. (A) Intraoperative image showing a smooth-surfaced, gelatinous mass approximately 4 × 2 cm being extracted through a transverse abdominal aortotomy (green arrow). (B) Macroscopic appearance of the excised mass, consistent with embolized cardiac myxoma, featuring a narrow stalk-like proximal end (yellow circle) and a bifurcated distal portion conforming to the aortic bifurcation. (For interpretation of the references to colour in this figure legend, the reader is referred to the web version of this article.)

Based on the imaging findings, the patient was diagnosed with occlusion of the abdominal aorta and bilateral common iliac arteries and was scheduled for surgical intervention. He was managed with intravenous heparin (150 IU/kg), vasodilators, and analgesics, which led to notable symptom improvement. Definitive surgical intervention was scheduled two days post-admission. Through a midline laparotomy, the abdominal aorta and bilateral iliac arteries were exposed and clamped. A transverse aortotomy was performed at the site of thrombotic occlusion, as previously identified on computed tomography imaging. This revealed a 4 × 2 cm gelatinous, smooth-surfaced mass obstructing the lumen (Fig. 2A). The structure was consistent with embolized cardiac myxoma, with a narrow stalk-like end and a bifurcated distal portion conforming to the iliac bifurcation (Fig. 2B).

Following excision, the vessels were flushed and restored to flow. The aortotomy was closed using Prolene sutures. Good pulsation was confirmed in the femoral and dorsalis pedis arteries. The abdominal wall was reapproximated in anatomical layers. Postoperatively, the patient recovered uneventfully with full restoration of limb function and perfusion.

**Fig. 3:**
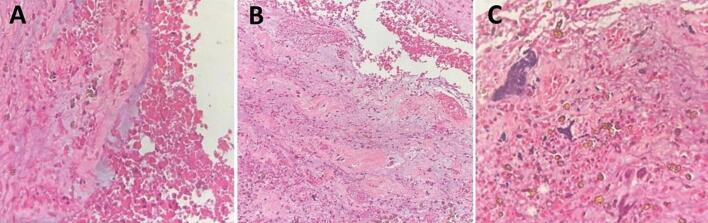
Histopathological features of the embolized cardiac myxoma (Hematoxylin & Eosin stain). (A) Low-power view showing abundant myxoid stroma with scattered hemorrhage and loosely arranged stellate cells. (B) Intermediate magnification demonstrating spindle- and polyhedral-shaped tumor cells embedded in mucinous matrix. (C) High-power view revealing clusters of tumor cells surrounding delicate capillary-like vessels with occasional foci of hemosiderin and microcalcification.

Postoperative echocardiography revealed a pedunculated mass in the left atrium originating from the interatrial septum. Histopathological analysis confirmed the diagnosis of cardiac myxoma, demonstrating mucinous stromal tissue with spindle and polyhedral cells arranged around delicate vasculature (Fig. 3A and B) and occasional calcifications (Fig. 3C).

The patient was discharged on postoperative day 7 and subsequently attended follow-up visits at 1 month, 3 months, and every 6 months thereafter. The most recent follow-up occurred at four years post-surgery. Postoperative echocardiography revealed a small stalk-like remnant in the left atrium, which was no longer detectable at 3- and 6-month follow-up studies, suggesting complete spontaneous regression. Unfortunately, echocardiographic images were not archived for documentation. The residual stalk in the left atrium exhibited no further growth, and vascular examination of the lower extremities showed no abnormalities.

## 3 Discussion

Atrial myxoma is the most common primary cardiac tumor, accounting for approximately 50 % of benign cardiac neoplasms, with the majority arising in the left atrium [3]. While histologically benign, these tumors can cause life-threatening complications through embolization or intracardiac obstruction. Embolic phenomena are reported in 30–40 % of patients and can affect cerebral, retinal, coronary, renal, or peripheral arteries depending on the size, mobility, and surface morphology of the tumor [5].

Abdominal aortic embolization due to atrial myxoma is exceedingly rare. Most reported embolic events involve smaller arteries, with only a few cases describing embolic obstruction at the level of the infrarenal abdominal aorta [6]. In our case, the patient presented with bilateral lower limb symptoms but retained limb perfusion due to well-developed collateral circulation. This atypical presentation underscores the importance of considering embolic etiologies even in the absence of classical ischemic signs.

CT angiography was essential in identifying the site and extent of the occlusion, and distinguishing embolism from other etiologies such as thrombosis or dissection [1]. The absence of arterial wall calcification and the morphology of the embolic material supported the diagnosis of embolization. Surgical exploration confirmed the presence of a gelatinous mass consistent with myxoma.

The standard treatment for atrial myxoma involves complete surgical excision, typically via median sternotomy under cardiopulmonary bypass. Immediate resection is recommended to prevent further embolic events and sudden cardiac death [7]. However, in this case, the residual stalk identified on initial echocardiography showed spontaneous regression and no progression or recurrence was noted on follow-up, which is unusual and rarely reported in the literature. This raises the possibility that, in selected patients where postoperative imaging confirms complete embolization of the tumor with no significant remnant, surgical excision of the primary cardiac lesion may not be immediately necessary. Nevertheless, careful monitoring is warranted, as spontaneous resolution is rare and the risk of recurrence or embolization persists.

Furthermore, this case highlights the importance of preoperative cardiac imaging. In our case, echocardiography was not performed before surgery, and the cardiac origin of the embolus was only suspected after surgical findings and confirmed by postoperative imaging. Earlier use of echocardiography may have altered the perioperative strategy and potentially prompted earlier cardiology consultation and planning for tumor resection if needed. Thus, echocardiography should be an integral part of the diagnostic workup in patients presenting with large vessel embolism of unknown origin.

Although rare, this case illustrates the need for high clinical suspicion and prompt multidisciplinary evaluation when encountering acute aortic occlusion without overt signs of ischemia. Echocardiography should be promptly employed in unexplained embolic cases to identify cardiac sources. In selected cases, close echocardiographic monitoring may be appropriate when the residual lesion shows signs of spontaneous resolution, though long-term data are lacking. Nonetheless, in this case the lack of echocardiographic imaging limits definitive confirmation.

## 4 Conclusions

Abdominal aortic embolization secondary to atrial myxoma is rare but life-threatening. Prompt diagnosis using CT angiography and echocardiography, followed by timely surgical management, is critical for survival. Long-term follow-up is essential due to the risk of recurrence.

## Abbreviations

CT: computed tomography
ECG: electrocardiogram

## Author contribution

TMN, MSL, CHN and MTD: Management of the case and preparing the manuscript. TMN, DQH, MTD: Management of the case and critical appraisal and review of the manuscript. DQH, CHN and MTD: Visualization. All authors read and approved the final manuscript.

## Ethical approval

This study was approved by the Institutional Review Board of the current institution.

## Guarantor

The-May Nguyen and Minh-Tung Do.

## Research registration number

It is not a “first in human” study.

## Declaration of Generative AI and AI-assisted technologies in the writing process

During the preparation of this work the authors used Paperpal in order to check grammar and writing. After using this tool/service, the authors reviewed and edited the content as needed and take full responsibility for the content of the publication.

## Funding

This research did not receive any specific funding.

## Consent to publish declaration

Written informed consent for publication has been obtained from the participant.

## Conflict of interest statement

The authors declare that there is no conflict of interest.
